# *SPO73* and *SPO71* Function Cooperatively in Prospore Membrane Elongation During Sporulation in *Saccharomyces cerevisiae*

**DOI:** 10.1371/journal.pone.0143571

**Published:** 2015-11-25

**Authors:** Emily M. Parodi, Joseph M. Roesner, Linda S. Huang

**Affiliations:** Department of Biology, University of Massachusetts Boston, Boston, Massachusetts, United States of America; Institute of Biology Valrose, FRANCE

## Abstract

In the yeast *Saccharomyces cerevisiae*, cells undergoing sporulation form prospore membranes to surround their meiotic nuclei. The prospore membranes ultimately become the plasma membranes of the new cells. The putative phospholipase Spo1 and the tandem Pleckstrin Homology domain protein Spo71 have previously been shown to be required for prospore membrane development, along with the constitutively expressed Vps13 involved in vacuolar sorting. Here, we utilize genetic analysis, and find that *SPO73* is required for proper prospore membrane shape and, like *SPO71*, is necessary for prospore membrane elongation. Additionally, similar to *SPO71*, loss of *SPO73* partially suppresses *spo1*Δ. Spo73 localizes to prospore membranes and complexes with Spo71. We also find that phosphatidylserine localizes to the prospore membrane. Our results suggest a model where *SPO71* and *SPO73* act in opposition to *SPO1* to form and elongate prospore membranes, while *VPS13* plays a distinct role in prospore membrane development.

## Introduction

In the budding yeast *Saccharomyces cerevisiae*, diploid cells can respond to starvation by triggering the developmental process of sporulation. During this process, the cell undergoes significant changes to both its ploidy and its cellular architecture [1–3]. The ability of the cell to properly complete sporulation is coupled to formation of prospore membranes, which become the plasma membranes for the future spores [4,5]. Prospore membrane growth is coordinated by multiple mechanisms to specify precise dynamic shapes that ensure encapsulation of genomic and cytosolic content, and also forms the template for spore wall morphogenesis [6–8]. Prospore membrane morphogenesis occurs in four stages: (i) initiation, (ii) elongation, (iii) rounding, and (iv) closure. While many genes have been linked to prospore membrane development, how these genes act together to regulate membrane morphogenesis is not fully understood.

*VPS13* encodes a vacuolar-protein family member [9,10]. Vps1 is expressed in vegetatitvely growing cells, upregulated during sporulation, and required for its successful completion [11–13]. Loss of *VPS13* results in the formation of tiny prospore membranes that frequently fail to capture nuclei [13,14]. *VPS13* has been proposed to act with *SPO71* [15], which encodes a tandem Pleckstrin-homology domain protein important for prospore membrane elongation [12,16].

Similar to *VPS13* and *SPO71*, the putative phospholipase-A2/B encoded by *SPO1* is upregulated during sporulation and required for its completion [11,12,17]. However, *spo1*Δ alleles exhibit a prospore membrane defect distinct from either *vps13*Δ or *spo71*Δ, as the loss of *SPO1* reduces the overall formation of prospore membranes, as assayed by a prospore membrane reporter, Spo20^51-91^[16]. Many *spo1*Δ cells fail to form prospore membranes, and instead exhibit clustering of phosphatidic acid throughout the cytoplasm. Those *spo1*Δ cells that do form prospore membranes have prospore membranes that grow inappropriately straight, producing a grossly elongated morphology [13,17]. *SPO1* has been proposed to be part of the sporulation membrane bending pathway, which acts to provide an inward bending force upon the prospore membrane [17]. Another gene required for sporulation, *SPO73*, encodes a Dysferlin domain protein [18, 19]. *SPO73* had previously been investigated for its role in prospore membrane development but was reported to be dispensable for proper prospore membrane shape [19].

We sought to determine how *SPO73* contributed to sporulation and found that, contrary to earlier reports, it is required for the proper formation of prospore membranes. We find that the requirement for *SPO73* in prospore membrane development is similar to that of *SPO71*, with both genes being required for prospore membrane elongation. Furthermore, we find that *SPO71* and *SPO73* act downstream of the early-acting prospore membrane gene *VPS13*, and that *SPO73*, *SPO71*, and *SPO1* have a complex genetic relationship necessary for sporulation.

## Materials and Methods

### Strains used in this study

All strains used in this study are derivatives of the highly efficient sporulating SK1 strain [20], and are listed in S1 Table. Gene knockouts were created using standard yeast genetic techniques [21]. *spo73*Δ isolates were constructed by replacing the *SPO73* open reading frame in a wild type MAT**a** strain, LH175 with either the *HIS3* ^*C*.*g*.^ gene amplified from pCgHIS (which contains the *Candida glabrata HIS3* gene) [16] to produce *spo73*::*HIS3* ^*C*.*g*.^, or the *LEU2* gene amplified from pCgLEU2 (which contains the *Candida glabrata LEU2 gene*) [22] to produce *spo73*::*LEU2* ^*C*.*g*.^. *SPO73-ENVY* was constructed by inserting the GFP variant, Envy, immediately before the stop codon of *SPO73* using PCR mediated recombination from PCR products amplified from pFA6a-link-Envy-SpHis5 [23]. Transformants were confirmed for proper tagging/gene replacement using PCR, and subsequently backcrossed to a MATα strain. MAT**a** and MATα segregants were verified using both auxotrophic marker identification and PCR genotyping and then mated to produce homozygous diploid strains, The *vps13*Δ homozygous diploid was generated by crossing HI28 [14] with the MATα strains LH899 and LH176, sporulating the heterozygotes, and dissecting haploids to generate haploids containing the *vps13*::*his*5+ allele with and without the *HTB2*-*mCherry* alleles. As with all strains, segregants were verified using auxtrophic marker identification and subsequent PCR confirmation.

### Plasmids

Plasmids used in this study are as follows: G20 (GFP-Spo20^51-91^) [13] and mTag2-BFP-Spo20^51-91^ [24] were used to visualize prospore membranes. Lact-C2-GFP-p416 (obtained from Addgene) [25] was used to detect phosphatidylserine localization. GFP-Spo14 [26] was used for Spo14 localization.

### Sporulation

Sporulation was performed as described previously [16]. For all assays involving sporulation efficiency, meiotic kinetics were monitored, and counts were only included for cultures that were undergoing sporulation efficiently, as assayed by having at least 50% of the cells entering meiosis by 8 hours post-sporulation induction. Meiosis was monitored by counting cells that had 1 nucleus, 2 nuclei, or > 2 nuclei, using either the fluorescently tagged Htb2 protein or DAPI staining.

### Fluorescence microscopy

All strains were imaged at 100x magnification through a 1.45 N.A. with the Axioskop Mot2 widefield microscope (Zeiss). Images were collected using an Orca-ER CCD camera (Hamamatsu) and Openlab 4.04 (Perkin Elmer) software. Image processing was performed using ImageJ1.46r (NIH), [27]. For prospore membrane analysis, multiple z-slices were summed to visualize all prospore membranes in each cell. Fluorescent images with were de-convolved using the Iterative Deconvolve plugin for ImageJ [28]. Prospore membrane measurements and statistical comparisons were performed as previously described [16].

### Phenotypic Assignment and Statistical Analysis

A minimum of three independent sporulations from each isolate was performed for all quantified phenotypes described. For comparison between genotypes, statistical comparisons were assessed using one-way analysis of variance and subsequent Tukey multiple comparison tests (GraphPad).

### Protein Immunoblotting

Protein lysates were prepared using trichloroacetic (TCA) denaturation, as described previously [29]. Precipitated proteins were resuspended in sample buffer [30], boiled for 5 minutes and separated via SDS-PAGE. Proteins were transferred onto polyvinylidene fluoride (GE Healthcare) and blocked using Odyssey PBS blocking buffer (LI-COR). Blots were probed with the following antibodies: mouse monoclonal 22C5D8 (Abcam) at 1:1000 for Pgk1 detection, mouse monoclonal JL-8 (BD Living Colors) at 1:1000 for GFP detection, rabbit polyclonal Ndt80 [31] at 1:1000 for Ndt80 detection and mouse monoclonal 9E10 (Covance) at 1:1000 for Myc detection, followed by Donkey Anti-Mouse IR Dye 800 CW or Donkey Anti-Rabbit IR Dye 680 RD (LI-COR) at 1:10000 as a secondary antibody. Blots were visualized on the Odyssey CLx Infrared Imaging System (LI-COR).

### Immunoprecipitation

Samples for immunoprecipitation were prepared from 60 OD_600_ of cells. Cell pellets were lysed in a MiniBeadBeater8 (Biospec) at 4°C with glass beads in IP buffer (150 mM KCl, 1 mM EDTA, 50 mM HEPES pH 7.6, 1 mM DTT and 0.5% Nonidet P-40) containing a mixture of protease and phosphatase inhibitors as previously described [30]. Lysates were clarified three times at 17,000 rcf at 4°C. Clarified lysates were mixed with 40 μl blocked agarose beads (Bulldog Bio), incubated at 4°C for 30 minutes. Beads were removed from the lysates by centrifugation at 500 rcf, and lysates were mixed with 20 μl of GFP-Trap A beads (Bulldog Bio) and incubated 2 hours at 4°C. GFP-Trap complexes were washed three times in IP buffer and re-suspended in 2x SDS-PAGE sample buffer, boiled for 5 minutes, separated by SDS-PAGE, and detected for proteins as described above.

## Results

### *SPO73* is required for prospore membrane elongation

**Fig 1:**
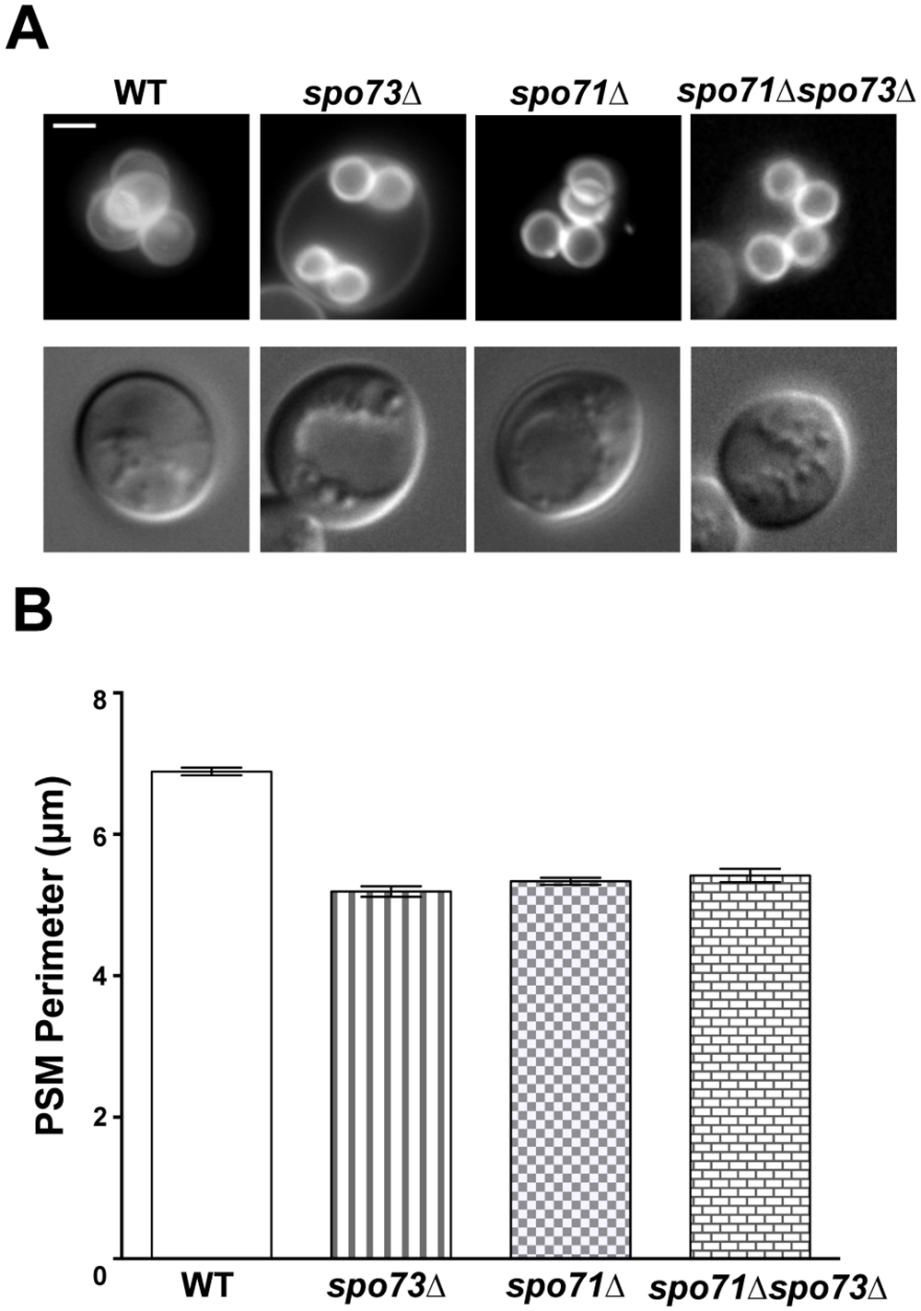
*SPO73* is necessary for proper prospore membrane size. (A) Images of characteristic prospore membranes (PSMs) in wild-type, *spo73*Δ, *spo71*Δ, and *spo71*Δ*spo73*Δ cells. Upper panel, PSMs marked with GFP-Spo20(^51–91^), lower panel, DIC. Scale bar, 2 μm. (B) Average PSM perimeters for genotypes shown in A. Error bars are standard error of the mean. Number of PSMs measured for each genotype: WT [319], *spo73*Δ [176], *spo71*Δ [257], and *spo71*Δ*spo73*Δ [100].

As *SPO73* resembled a regulator of the prospore membrane morphogenesis *SPO71* both in its expression profile and its dispensability for meiosis [11,12,18,19], we examined prospore membranes in *spo73*Δ cells. Prospore membrane analysis using the prospore membrane marker G20 (GFP-Spo20^51-91^) [13], shows that *SPO73* is required for the formation of properly sized prospore membranes. *spo73*Δ cells make prospore membranes that are terminally smaller than those made in wild-type cells (Fig 1A). We see this similar phenotype using two independently constructed alleles of *spo73* (*spo73*::*HIS3* ^*C*.*g*.^ and *spo73*::*LEU2* ^*C*.*g*.^). The mean (+/- SEM) prospore membrane perimeter sizes for the *spo73*::*HIS3* ^*C*.*g*.^ allele (5.11+/-0.08 μm) was not statistically distinct from that of the *spo73*::*LEU2* ^*C*.*g*.^ allele, (5.46+/-0.15 μm; *p-*value ~0.2). Intriguingly, the *spo73*Δ prospore membrane phenotype is similar to that seen in cells lacking *SPO71* (Fig 1B). Like *spo71*Δ cells, *spo73*Δ cells fail to exhibit the characteristic elongated tube phase of prospore membrane development. The elongated prospore membranes seen in wild-type cells were not seen in either single mutant or the double mutant.

To test whether *SPO73* and *SPO71* may act in the same or parallel pathways, we assessed the prospore membrane shape and size in a *spo71*Δ *spo73*Δ double mutant. We found that loss of both genes resulted in prospore membranes that are equivalent in size and shape as compared to those formed in either single mutant (Fig 1). The prospore membrane perimeters among mutants are not statistically different from each other, while the *spo71*Δ and *spo73*Δ single mutants and the *spo71*Δ *spo73*Δ double mutants are all statistically distinct from wild type (Tukey HSD, alpha = 0.01). This similar and not additive phenotype is consistent with *SPO73* and *SPO71* functioning within the same pathway for prospore membrane elongation.

### Spo73 localizes to prospore membranes and complexes with Spo71

**Fig 2:**
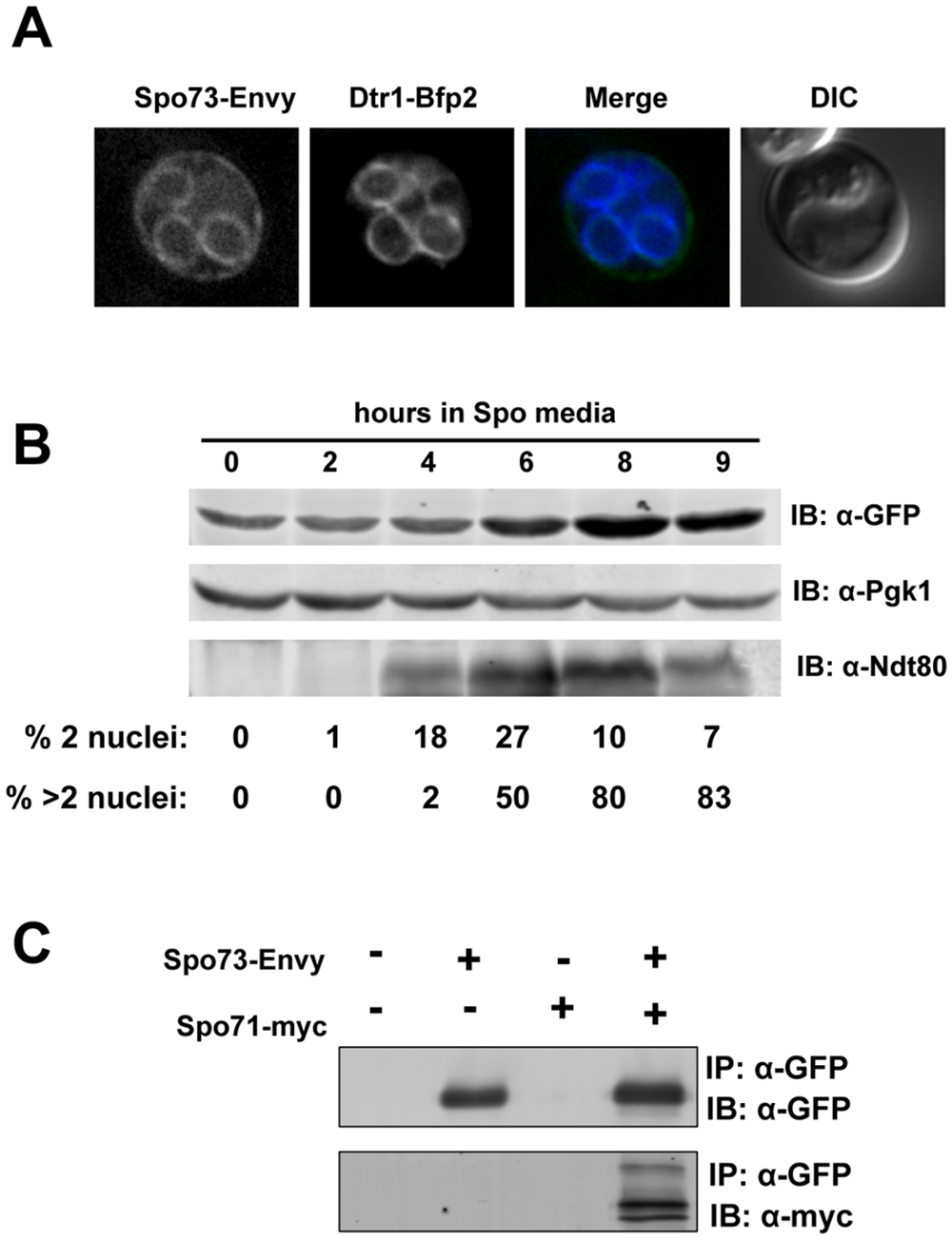
Spo73 localizes to prospore membranes. (A) Spo73-Envy and Dtr1-BFP imaged in live cells. Spo73-Envy colocalized with the prospore membrane localized Dtr1-BFP. (B) Spo73-Envy expression throughout sporulation. 0hr indicates transfer from sporulation priming media (YPA) into sporulation media (SPO). Pgk1 protein abundance serves as a loading control. Ndt80 expression coincides with expression of mid-sporulation genes, and protein abundance serves as a monitor for progression through sporulation. Meiotic counts were conducted from the same cultures used for protein isolation; 200 cells from each time point were counted. Nuclei were visualized using *HTB2-mCherry*. % 2 nuclei represents cells entering meiosis and may include cells that will sporulate as dyads, %>2 nuclei represents cells who have progressed into MII. (D) Spo71-myc coimmunoprecipitates with Spo73-Envy. Sporulated cells from wild-type (LH177), Spo71-myc (LH901), Spo73-Envy (LH938), and Spo71-myc+Spo73-Envy (LH1039) strains were immunoprecipitated with GFP and probed for Spo713 using and anti-GFP antibody and for Spo71 using an anti-myc antibody.

To assess *SPO73* localization in living cells, we tagged the endogenous *SPO73* at its C-terminus using the optimized GFP, Envy [23]. Strains homozygous for the *SPO73-ENVY* allele sporulate at wild-type levels, confirming that the tagged allele is functional. Using this tagged allele, we see Spo73 colocalizing with the prospore membrane localized Dtr1 [32] (Fig 2A). Thus, Spo73 localizes to the prospore membrane, consistent with a role in its development.

Western blotting indicated that the Spo73-Envy protein was produced during sporulation, with levels peaking when the majority of cells have completed meiosis (Fig 2B). Because our genetic analysis suggests that *SPO71* and *SPO73* function together, we tested whether the two proteins encoded by these genes physically interact. Immunoprecipitation of Spo73-Envy coimmunoprecipitates Spo71-myc (Fig 2C). As Spo71 was reported as localizing to the prospore membrane [15], this coimmunoprecipitation result is consistent with both proteins localizing to prospore membranes.

### *SPO71*, *SPO73* and *SPO1* have a complex genetic relationship

**Fig 3:**
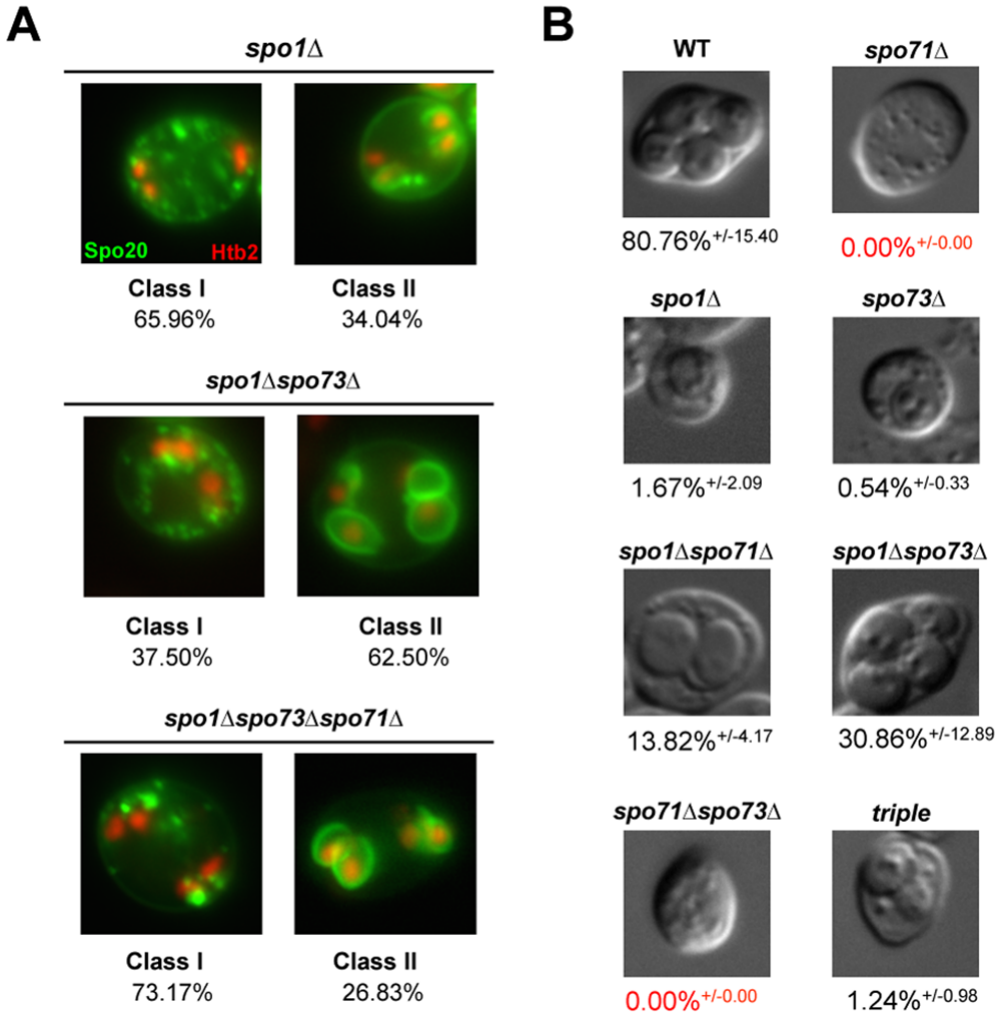
*SPO73* acts in opposition to *SPO1*, similar to *SPO71*. (A) The prospore membrane formation defect in *spo1*Δ cells is partially suppressed by the *spo73*Δ allele. Class I indicates failure to form 1 or more PSM, Class II indicates 1 or more PSMs formed. Number of asci scored for each genotype: *spo1*Δ [47], *spo73*Δ*spo1*Δ [48], and *spo73*Δs*po71*Δ*spo1*Δ [41]. (B) Representative images of the types of refractile structures present in mutants. Images are from cells 24–36 hours post-sporulation induction. % indicates frequency of refractile structures. Number of cells scored for each genotype: wild type [900], *spo71*Δ [938], *spo1*Δ [1,134], *spo73*Δ [1,426], s*po1*Δ*spo71*Δ [1,031], *spo1*Δ*spo73*Δ [1,094], and triple deletion (*spo73*Δs*po71*Δ*spo1*Δ) [945].

Prior work has found that *SPO71* acts antagonistically to the putative phospholipase *SPO1* during prospore membrane development [16]. As *SPO71* and *SPO73* have similar effects on prospore membrane size, we examined whether *SPO73* might also antagonize *SPO1*. Previously, we have seen that loss of *SPO1* results in a marked decrease in the formation of prospore membranes, with most cells exhibiting a clustering of prospore membrane materials throughout their cytoplasm [16]. We now find that, that similar to *SPO71*, the loss of *SPO73* also improves prospore membrane development in cells lacking *SPO1* (Fig 3A). Thus, *SPO73* and *SPO71* have a similar antagonistic relationship to *SPO1*.

In the simplest scenario, one might anticipate that the simultaneous removal of both *SPO73* and *SPO71* activity would cause a similar or an even greater suppression of the *spo1Δ* defect. Surprisingly, the *spo1*Δ*spo71*Δ*spo73*Δ triple mutant exhibits a prospore membrane formation defect most similar to the *spo1*Δ single mutant (Fig 3A). Thus *SPO71* and *SPO73* do not appear to form a simple linear pathway.

To further confirm these findings, we sought a separate quantitative measure of spore formation. While most *spo1*Δ cells are unable to form spores [33, 34], we do see that *spo1*Δ cells produce a low-level (1.67%) of refractile spore-like structures twenty-four to thirty-six hours following induction of sporulation, although they do not typically produce four spores within the ascus (Fig 3B). Refractile structures appear during spore development as the outer prospore membrane bilayer is removed and the outer chitosan and dityrosine layers are being deposited [19]. While it is important to note that the appearance of refractile structures does not necessarily mean the completion of spore development, it provided a tool for further confirming the genetic relationships among *SPO1*, *SPO71*, and *SPO73*. Visual analysis of single mutants revealed that like *spo1*Δ, loss of *SPO73* also corresponded to a low-level (0.54%) appearance of refractile spore-like structures, while loss of *SPO71* never resulted in the appearance of such structures (Fig 3B). All refractile spore-like structures we counted were observed to surround a nucleus, as would be expected for a maturing spore. When we examine *spo71*Δ*spo73*Δ double mutant, no refractile spore-like structures were observed, similar to the *spo71Δ* mutant.

Consistent with the antagonistic relationship between *SPO1* and either *SPO71* or *SPO73* that we observed by analyzing prospore membrane development, loss of *SPO73* or *SPO71* also suppresses *spo1*Δ for the refractile spore phenotype. *spo1*Δ*spo71*Δ (13.82%) and *spo1*Δ*spo73*Δ (30.86%) mutants exhibited a significant increase in the appearance of refractile spore-like structures compared to any single mutant. Furthermore, the triple mutant, *spo1*Δ *spo71*Δ *spo73*Δ formed few refractile structures (1.24%), similar to the *spo1*Δ single mutant. This triple mutant phenotype is consistent with what we see when we examine the prospore membrane, where we see that the *spo1*Δ*spo71*Δ*spo73*Δ mutant produces a prospore membrane phenotype most similar to *spo1*Δ mutant (Fig 3A). These data confirm that while the loss of either *SPO71* or *SPO73* can compensate to some extent for the loss of *SPO1*, loss of both *SPO71* and *SPO73* eliminates this effect.

### Phosphatidylserine and Spo14 localization is not altered in *spo73*, *spo71*, or *spo1* mutants

**Fig 4:**
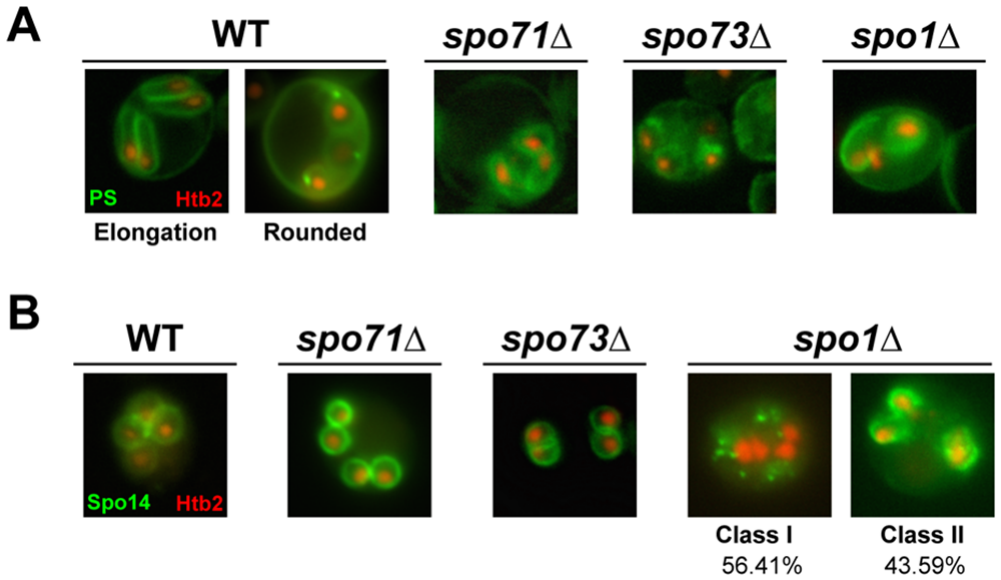
Phosphatidylserine and Spo14 localization confirm prospore membrane phenotypes. (A) Localization of phosphatidylserine (PS) in wild-type, *spo71*Δ, *spo73*Δ and *spo1*Δ cells. PS was detected using Lact-C2-GFP-p416 [3]. Both elongated and rounded prospore membrane stages are shown for wild-type cells; elongated prospore membranes are not typically seen in *spo71*Δ and *spo73*Δ cells (B) Localization of Spo14 in wild-type, *spo71*Δ, *spo73*Δ and *spo1*Δ cells. Spo14 was detected using GFP-Spo14 [26]. For *spo1*Δ, Class I represents cells without prospore membranes; Class II cells represents cells that make at least one prospore membrane, like in Fig 3. n = 39.

Previous studies suggested that the prospore membrane marker GFP-Spo20^51-91^ may bind to phosphatidic acid [35]. However, recent work has demonstrated that this construct can interact with multiple anionic lipids [36]. Because we had assayed prospore membranes using GFP-Spo20^51-91^, it is possible that the prospore membrane phenotypes we see may be due to mislocalization of one or multiple GFP-Spo20^51-91^ interacting anionic lipids, rather than a complete loss in formation of prospore membranes. Thus, we assayed the localization of phosphatidylserine, an anionic phospholipid found in eukaryotic plasma membranes. While phosphatidylserine is present in a polarized fashion at the bud neck and bud cortex of vegetatively growing yeast cells [37, 38], its localization during sporulation has not been previously reported. Using the phosphatidylserine reporter Lact-C2-GFP [25], we found that phosphatidylserine localizes to the developing prospore membrane (Fig 4A). We also see that phosphatidylserine localizes to the aberrant prospore membranes in the *spo1*Δ, *spo71*Δ, and *spo73*Δ mutants. These results suggest that the prospore membrane defect we see using the phosphatidic acid sensor does reflect a prospore membrane defect and not just a problem with the localization of certain anionic lipids.

To further assess whether the observed defects reflect a membrane morphogenesis rather than lipid biogenesis defect, we examine the localization of Spo14, a phospholipase-D that catalyzes the production of phosphatidic acid from phosphatidylcholine, and localizes to the prospore membrane [26,39]. We see Spo14 localization as expected, localizing in developmentally appropriate patterns along the prospore membrane (Fig 4B). Taken together, the prospore membrane marker GFP-Spo20^51-91^, the phosphatidylserine marker Lact-C2-GFP, and GFP-Spo14 all show that *spo73* mutants have a prospore membrane defect.

### *vps13*Δ mutants have a more severe prospore membrane defect compared to *spo71*Δ and *spo73*Δ mutants

**Fig 5:**
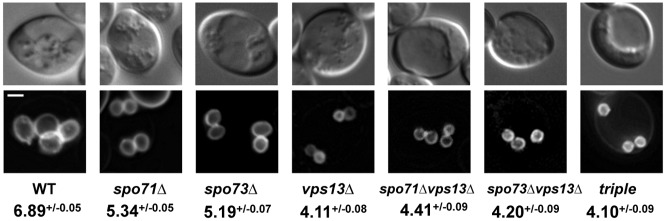
Loss of *VPS13* reduces prospore membrane size more severely than loss of *SPO71* or *SPO73*. Top photos: DIC images. Bottom photos: PSM visualized using the Spo20 PSM marker, as in Fig 1. PSM average perimeters in μm +/- SEM are given below the genotypes. The number of PSM measured for each genotype are: wild type (319), *spo71*Δ (257), *spo73*Δ (176), *vps13*Δ (110), *spo71*Δ *vps13*Δ (136), *spo73*Δ *vps13*Δ (101), triple (*vps13*Δ *spo73*Δ*spo71*Δ) (100). Scale bar, 2 μm.

The phenotypes of *spo71*Δ and *spo73*Δ mutants suggest that *SPO71* and *SPO73* act during the elongation phase of prospore membrane development. *VPS13* has previously been shown to be required for the formation of prospore membranes of appropriate size [13], and has been proposed to act as a partner for *SPO71* [15]. We find that the prospore membrane defect of *vps13*Δ cells is more severe than that observed in *spo71*Δ or *spo73*Δ cells (Fig 5), with a comparison of perimeter sizes among mutants revealing that *vps13*Δ mutant perimeter sizes are statistically distinct (Tukey HSD, alpha = 0.01). *vps13*Δ mutants produce tiny prospore membranes that are smaller than those seen in *spo73* and *spo71*. Prospore membranes in *vps13*Δ mutants lacking either or both *SPO73* and *SPO71* exhibit tiny prospore membranes, like those observed in *vps13*Δ single mutants (Fig 5). Although Vps13 has been suggested to perform at least one of its functions with Spo71 [15], these epistasis results suggest that Vps13 has a role in prospore membrane development that is distinct from Spo71 and Spo73.

## Discussion

Taken together, our data show that *SPO73*, an essential regulator of sporulation whose role in the process was previously unclear, acts to promote prospore membrane elongation. The effect of *SPO73* on prospore membrane elongation resembles those previously reported for the Pleckstrin Homology domain protein *SPO71* [16]. We further find that Spo73 localizes to prospore membranes, similar to the reported localization of Spo71 at elongated and rounded prospore membranes [14]. Our localization of Spo73 differs from the previously reported cytoplasmic and punctate localization [19]; however, those previous studies determined Spo73 localization in fixed cells using immunofluorescence techniques which are more prone to artifacts and complicate stage-specific localization. The interaction of *SPO71* and *SPO73* is also seen biochemically, as Spo71 and Spo73 can be coimmunoprecipitated from sporulating cells. Our data also show that while *SPO73*, like *SPO71*, acts antagonistically to the putative phospholipase *SPO1* during spore morphogenesis, removing both *SPO73* and *SPO71* eliminates the effect, suggesting that perhaps the balance of *SPO73* and *SPO71* activity is important. Finally, we our results suggest that the vacuolar protein *VPS13* exerts a broader impact on the process of sporulation than either *SPO73* or *SPO71*, suggesting an additional, different role for *VPS13* from *SPO73 and SPO71*.

### *SPO73* and *SPO71* function during prospore membrane elongation, distinct from *VPS13*

The dynamic shapes of the developing prospore membrane provide important clues to determine the stage(s) in which regulators of prospore membrane morphogenesis function. This work suggests that *SPO73* and *SPO71* act together to promote prospore membrane elongation, as the *spo71*Δ and *spo73*Δ mutants show defects in prospore membrane elongation, as the *spo71*Δ*spo71*Δ double mutant phenotype is no worse than either single mutant, and as Spo73 and Spo71 physically interact, as assayed by coimmunoprecipitation. Interestingly, the *spo71*Δ phenotype is more severe than that seen for *spo73*Δ, as assayed by the appearance of refractile spore-like structures, suggesting that *SPO71* plays a greater role than *SPO73*.

Our results suggest that *SPO71* and *SPO73* play a distinct role in prospore membrane development from *VPS13*. The extreme reduction in prospore membrane size exhibited by *vps13*Δ cells seen by both others [14] and ourselves suggests that loss of *VPS13* causes prospore membranes to prematurely arrest during development. These results are consistent with *VPS13* acts during an initial growth phase of the prospore membrane, when the membrane starts to grow from the meiotic outer plaque and when it is crucial for the cell to determine the direction of membrane growth relative to the nucleus. From our results, it is possible that *SPO71* and *SPO73* act as negative regulators of *VPS13*, that *SPO71* and *SPO73* act downstream of *VPS13*, or, merely that *SPO71* and *SPO73* work independently of *VPS13*. Further experiments are needed to clarify the relationship between these genes.

### Opposing pathways promote sporulation

Our study demonstrates that *SPO73* and *SPO71* exhibit similar genetic relationships with *SPO1* during sporulation. We see this relationship using two assays: by the examination of prospore membrane sensors during prospore membrane morphogenesis, and by examining the formation of refractile structures following sporulation. We had previously demonstrated that clustering of prospore membrane materials that occurs in the majority of *spo1*Δ cells is suppressed by loss of *SPO71*. Here, we observe a similar phenomenon, where *spo73*Δ acts as a suppressor of *spo1*Δ, promoting the formation of prospore membranes and reducing inappropriate phosphatidic acid clustering. When we assay the formation of refractile spore-like structures, we find that both *spo1*Δ*spo71*Δ and *spo1*Δ*spo73*Δ mutants exhibited a significant increase in the appearance of refractile structures compared to all of the single mutants. Furthermore, the refractile structures seen in the double mutants appear much closer to wild-type spores, as compared to the structures seen in either the *spo1*Δ or *spo73*Δ mutant, further consistent with their antagonistic effects on *SPO1*.

Our analysis of the *spo1*Δ*spo71*Δ*spo73*Δ triple mutant yielded a surprising result, where the triple mutant was more defective than the *spo1*Δ*spo71*Δ and *spo1*Δ*spo73*Δ double mutants. The triple mutant exhibited a prospore membrane formation defect most similar to the *spo1*Δ single mutant. One possible explanation for this surprising result is that *spo71*Δ*spo73*Δ is more defective than either single mutant in some fashion. As *SPO71* activity is present in the *spo1*Δ*spo73*Δ double mutant and *SPO73* activity is present in the *spo1*Δ*spo71*Δ double mutant, these activities work to produce prospore membranes. However, in the *spo1*Δ*spo71*Δ*spo73*Δ triple mutant, this balance of defects is destroyed, creating a more severe defect.

## Supporting Information

S1 Table *S*. *cerevisiae* strains used in this study. (PDF)
