# Supplementary material for: SPO73 and SPO71 Function Cooperatively in Prospore Membrane Elongation During Sporulation in Saccharomyces cerevisiae
Source: PLoS One. 2015 Nov 25;10(11):e0143571. doi: 10.1371/journal.pone.0143571 (PMC4659569; doi:10.1371/journal.pone.0143571)
Supplement: S1 Table — (PDF) [file pone.0143571.s001.pdf]

S1 Table. *S. cerevisiae* strains used in this study

Strain	Genotype	Source
LH175	MAT**a** *ho::hisG lys2 ura3 leu2 his3 trp1ΔFA*	[30]
LH176	MATα *ho::hisG lys2 ura3 leu2 his3 trp1ΔFA*	[30]
LH177	MAT**a**/MATα *ho::hisG/ho::hisG lys2/lys2 ura3/ura3 leu2/leu2 his3/his3 trp1ΔFA/trp1ΔFA*	[30]
HI28	MAT**a** *his3ΔSK hoΔ::LYS2 leu2 lys2 trp1::hisG ura3 vps13::his5+*	[13]
LH899	MATα *ho::hisG lys2 ura3 leu2 his3 trp1ΔFA HTB2-mCherry-TRP1*$^{C.g.}$	[16]
LH901	MAT**a**/MATα *ho::hisG/ho::hisG lys2/lys2 ura3/ura3 leu2/leu2 his3/his3 trp1ΔFA/trp1ΔFA SPO71-13xMYC-TRP1/ SPO71-13xMYC-TRP1*	[16]
LH902	MAT**a**/MATα *ho::hisG/ho::hisG lys2/lys2 ura3/ura3 leu2/leu2 his3/his3 trp1ΔFA/trp1ΔFA HTB2-mCherry-TRP1*$^{C.g.}$*/HTB2-mCherry-TRP1*$^{C.g.}$	[16]
LH903	MAT**a**/MATα *ho::hisG/ho::hisG lys2/lys2 ura3/ura3 leu2/leu2 his3/his3 trp1ΔFA/trp1ΔFA HTB2-mCherry-URA3*$^{K.l.}$*/HTB2-mCherry-URA3*$^{K.l.}$	[16]
LH904	MAT**a**/MATα *ho::hisG/ho::hisG lys2/lys2 ura3/ura3 leu2/leu2 his3/his3 trp1ΔFA/trp1ΔFA spo71::TRP1*$^{C.g.}$*/spo71::TRP1*$^{C.g.}$ *HTB2-mCherry-TRP1*$^{C.g.}$*/HTB2-mCherry-TRP1*$^{C.g.}$	[16]
LH914	MAT**a**/MATα *ho::hisG/ho::hisG lys2/lys2 ura3/ura3 leu2/leu2 his3/his3 trp1ΔFA/trp1ΔFA spo1::HIS3/ spo1::HIS3 HTB2-mCherry-TRP1*$^{C.g.}$*/HTB2-mCherry-TRP1*$^{C.g.}$	[16]
LH915	MAT**a**/MATα *ho::hisG/ho::hisG lys2/lys2 ura3/ura3 leu2/leu2 his3/his3 trp1ΔFA/trp1ΔFA spo71::TRP1*$^{C.g.}$*/spo71::TRP1*$^{C.g.}$ *spo1::HIS3/ spo1::HIS3 HTB2-mCherry-TRP1*$^{C.g.}$*/HTB2-mCherry-TRP1*$^{C.g.}$	[16]
LH932	MAT**a**/MATα *ho::hisG/ho::hisG lys2/lys2 ura3/ura3 leu2/leu2 his3/his3 trp1ΔFA/trp1ΔFA spo73:: HIS3/ spo73:: HIS3 HTB2-mCherry-TRP1*$^{C.g.}$*/HTB2-mCherry-TRP1*$^{C.g.}$	This Study
LH933	MAT**a**/MATα *ho::hisG/ho::hisG lys2/lys2 ura3/ura3 leu2/leu2 his3/his3 trp1ΔFA/trp1ΔFA spo73:: HIS3/ spo73:: HIS3 spo1::HIS3/ spo1::HIS3 HTB2-mCherry-TRP1*$^{C.g.}$*/HTB2-mCherry-TRP1*$^{C.g.}$	This Study
LH934	MAT**a**/MATα *ho::hisG/ho::hisG lys2/lys2 ura3/ura3 leu2/leu2 his3/his3 trp1ΔFA/trp1ΔFA spo73:: HIS3/ spo73:: HIS3 spo71:: TRP1*$^{C.g}$ */ spo71:: TRP1*$^{C.g}$ *HTB2-mCherry-TRP1*$^{C.g.}$*/HTB2-mCherry-TRP1*$^{C.g.}$	This Study
LH935	MAT**a**/MATα *ho::hisG/ho::hisG lys2/lys2 ura3/ura3 leu2/leu2 his3/his3 trp1ΔFA/trp1ΔFA spo71:: TRP1*$^{C.g}$ */ spo71:: TRP1*$^{C.g}$ *spo73:: HIS3* $^{C.g.}$*/ spo73:: HIS3* $^{C.g.}$ *spo1::HIS3/ spo1::HIS3 HTB2-mCherry-TRP1*$^{C.g.}$*/HTB2-mCherry-TRP1*$^{C.g.}$	This Study
LH936	MAT**a**/MATα *ho::hisG/ho::hisG lys2/lys2 ura3/ura3 leu2/leu2 his3/his3 trp1ΔFA/trp1ΔFA vps13:: HIS3/ vps13:: HIS3 HTB2-mCherry-TRP1*$^{C.g.}$*/HTB2-mCherry-TRP1*$^{C.g.}$	This Study
LH937	MAT**a**/MATα *ho::hisG/ho::hisG lys2/lys2 ura3/ura3 leu2/leu2 his3/his3 trp1ΔFA/trp1ΔFA vps13:: HIS3/ vps13:: HIS3 spo71:: TRP1*$^{C.g}$ */ spo71:: TRP1*$^{C.g}$ *HTB2-mCherry-TRP1*$^{C.g.}$*/HTB2-mCherry-TRP1*$^{C.g.}$	This Study

LH938	MAT**a**/MATα *ho::hisG/ho::hisG lys2/lys2 ura3/ura3 leu2/leu2 his3/his3 trp1ΔFA/trp1ΔFA SPO73-Envy- SpHis5/ SPO73-Envy- SpHis5 HTB2-mCherry-TRP1*[C.g.]*/HTB2-mCherry-TRP1*[C.g.]	This Study
LH1035	MAT**a**/MATα *ho::hisG/ho::hisG lys2/lys2 ura3/ura3 leu2/leu2 his3/his3 trp1ΔFA/trp1ΔFA vps13:: HIS3/ vps13:: HIS3 spo73:: HIS3*[C.g] */ spo73::HIS3*[C.g] *HTB2-mCherry-TRP1*[C.g.]*/HTB2-mCherry-TRP1*[C.g.]	This Study
LH1036	MAT**a**/MATα *ho::hisG/ho::hisG lys2/lys2 ura3/ura3 leu2/leu2 his3/his3 trp1ΔFA/trp1ΔFA vps13:: HIS3/ vps13:: HIS3 spo71:: TRP1*[C.g] */ spo71:: TRP1*[C.g] *spo73:: HIS3*[C.g] */ spo73::HIS3*[C.g] *HTB2-mCherry-TRP1*[C.g.]*/HTB2-mCherry-TRP1*[C.g.]	This Study
LH1037	MAT**a**/MATα *ho::hisG/ho::hisG lys2/lys2 ura3/ura3 leu2/leu2 his3/his3 trp1ΔFA/trp1ΔFA spo73:: LEU2*[C.g.]*/ spo73:: LEU2*[C.g.] *HTB2-mCherry-TRP1*[C.g.]*/HTB2-mCherry-TRP1*[C.g.]	This Study
LH1038	MAT**a**/MATα *ho::hisG/ho::hisG lys2/lys2 ura3/ura3 leu2/leu2 his3/his3 trp1ΔFA/trp1ΔFA SPO73-Envy-SpHis5/ SPO73-Envy-SpHis5 Dtr1-BFP-KAN/ Dtr1-BFP-KAN*	This Study
LH1039	MAT**a**/MATα *ho::hisG/ho::hisG lys2/lys2 ura3/ura3 leu2/leu2 his3/his3 trp1ΔFA/trp1ΔFA SPO71-13xMYC-TRP1/ SPO71-13xMYC-TRP1 SPO73-Envy-SpHis5/ SPO73-Envy-SpHis5*	This Study
